# 

**Published:** 1860-12

**Authors:** 


### CONTENTS

OF THE

### Chicago Medical Journal for December, 1860

### ORIGINAL COMMUNICATIONS.

ARTICLE 1—Experiments with Iodine. By B. Woodward, M. D. .... 697

ARTICLE 2—Cases Successfully treated with Tincture of Iodine. By E. Nichols .... 701

ARTICLE 3—Diptheria. By R. B. Ray, M. D .... 704

ARTICLE 4—Case of Tetanus from very Slight Injury. By D. W. Hand, M. D .... 706

### EDITORIAL.

Introductory Address to the Class of Rush Medical College .... 729

Book Notices .... 745, 746

Proceedings of the DeWitt County Medical Society .... 747

The Great Medical Slander Suit .... 749

The Gazette and Dr. Holmes .... 750

Transactions of the Illinois State Medical Society .... 750

Rush Medical College .... 751

Sectionalism .... 752

Remarkable Controversy .... 752

Medical Department, Lind University .... 752

### SELECTED.

Extracts from an Essay on Diptheria and Diptheritic Affections .... 709

Periodicity as a Character of Disease .... 721

Treatment of Diptheria .... 721

The Use of Instruments in Obstetric Practice .... 726

Carelessness on the part of Correspondents .... 728

Removal of the Tongue .... 728

